# Characterization of Acyl-CoA Oxidases from the Lipolytic Yeast *Candida aaseri* SH14

**DOI:** 10.4014/jmb.2205.05029

**Published:** 2022-06-06

**Authors:** Zool Hilmi Ibrahim, Jung-Hoon Bae, Bong Hyun Sung, Mi-Jin Kim, Ahmad Hazri Ab Rashid, Jung-Hoon Sohn

**Affiliations:** 1 Synthetic Biology Research Center, Korea Research Institute of Bioscience and Biotechnology (KRIBB), Daejeon 34141, Republic of Korea; 2 Department of Biosystems and Bioengineering, KRIBB School of Biotechnology, Korea University of Science and Technology (UST), Daejeon 34113, Republic of Korea; 3 Industrial Biotechnology Research Centre, SIRIM Berhad, No.1, Persiaran Dato’ Menteri, Section2, 40700, Shah Alam, Selangor, Malaysia

**Keywords:** Acyl-CoA oxidase, lipolytic yeast, *Candida aaseri*, β-oxidation, substrate specificity

## Abstract

The lipolytic yeast *Candida aaseri* SH14 contains three Acyl-CoA oxidases (ACOXs) which are encoded by the *CaAOX2*, *CaAOX4*, and *CaAOX5* genes and catalyze the first reaction in the β-oxidation of fatty acids. Here, the respective functions of the three *CaAOX* isozymes were studied by growth analysis of mutant strains constructed by a combination of three *CaAOX* mutations in minimal medium containing fatty acid as the sole carbon source. Substrate specificity of the *CaAOX* isozymes was analyzed using recombinant *C. aaseri* SH14 strains overexpressing the respective genes. *CaAOX2* isozyme showed substrate specificity toward short- and medium-chain fatty acids (C6-C12), while *CaAOX5* isozyme preferred long-chain fatty acid longer than C12. *CaAOX4* isozyme revealed a preference for a broad substrate spectrum from C6-C16. Although the substrate specificity of *CaAOX2* and *CaAOX5* covers medium- and long-chain fatty acids, these two isozymes were insufficient for complete β-oxidation of long-chain fatty acids, and therefore *CaAOX4* was indispensable.

## Introduction

The lipolytic yeast *Candida aaseri* SH14, isolated from the compost of oil palm empty fruit bunches, can utilize various fatty acids and long-chain alkanes as a sole carbon source and is resistant to high concentrations of organic acids [1]. The capability to breakdown hydrophobic substrate may explain its isolation from oil palm agricultural waste. Therefore, this strain was considered a microbial factory for the production of bio-based chemicals from plant oils [2]. In mammalian cells, the biodegradation of fatty acids occurs mainly by β-oxidation in mitochondria and peroxisomes, whereas this event is only present in peroxisomes of yeasts [3, 4]. The breakdown of fatty acid is begun with the formation of fatty acyl-CoA by the fatty acyl-CoA ligase, which catalyzes the pre-step reaction for β-oxidation. The β-oxidation of yeast involves the sequential reaction of four enzymes: acyl-CoA oxidase (ACOX), enoyl-CoA hydratase, 3-hydroxyacyl-CoA dehydrogenase, and 3-ketoacyl-CoA thiolase [4, 5]. ACOX catalyzes the limiting step of peroxisomal β-oxidation by converting acyl-CoA to 2- trans-enoyl-CoA [6].

ACOX has been isolated from various organisms, including microbes [7-12], plants [13-16], and also mammals [17-20]. Most organisms contain several isozymes having different substrate specificities to utilize various carbon chain-length fatty acids as carbon sources. For example, *Saccharomyces cerevisiae* [8] and *Aspergillus nidulans* [21] contain one ACOX, while *Candida tropicalis* [10] and *Yarrowia lipolytica* [22] have five and six isoforms with different substrate specificities.

In a previous study, we identified three genes encoding ACOX (*CaAOX2*, *CaAOX4*, and *CaAOX5*) from the *C. aaseri* SH14 genome on the basis of sequence homology. A β-oxidation mutant completely lacking ACOX activity was directly constructed by simultaneous disruption of six copies of ACOX genes in diploid cells using the CRISPR-Cas9 system; however, the functional differences between these isozymes were not determined [2]. In this study, we have evaluated the ACOX activity of the three isozymes by in vivo study of the respective mutants to understand the utilization of fatty acid in *C. aaseri* SH14.

## Materials and Methods

### Strains, Chemicals and Medium

*C. aaseri* SH14 (*ura3*) [2], and their mutant strains were cultured in YPD (1% yeast extract, 2% peptone, and 2%glucose). Selection of URA+ transformants was performed on a synthetic complete medium lacking uracil (SC-ura; 0.67% yeast nitrogen base without amino acids, 0.077% ura dropout supplement, 2% glucose, and 2% agar). For screening of *CaAOX* mutants, YPD supplemented with 20 μg/ml nourseothricin (NTC, Sigma Chemicals Co., St. USA) was used. For *C. aaseri* SH14 growth study, minimal media supplemented with different carbon source was used. The composition of the media used in this study was as follows: standard minimal glucose medium (SMD) [1.7g of yeast nitrogen base without amino acid, and 20 g of glucose], minimal methyl laurate medium (SML) [1.7g of yeast nitrogen base without amino acid, and 1% (v/v) of methyl laurate (TCI Chemicals, Tokyo, Japan)], minimal oleic acid medium (SMO) [1.7 g of yeast nitrogen base without amino acid, and oleic acid (TCI Chemicals)]. All media were adjusted to pH 7.0 with NaOH. *Escherichia coli* DH5α [F-lacZΔM15 hsdR17(r-m-) gyrA36] was used for general recombinant DNA techniques. Cell growth was measured by spectrophotometer at 600 nm. Cells were collected by centrifugation and washed with sterile distilled water to eliminate fatty acid before measurement. Restriction enzymes were purchased from New England Biolabs (USA). DNA purification was performed using the Wizard SV Gel, and a PCR Clean-Up System (Promega, USA).

### Construction of CRISPR-Cas9 Vector for Disruption of Acyl-CoA Oxidase Isozyme Genes

The episomal CRISPR-Cas9 vector, pAN-Cas9, developed for gene manipulation of diploid yeast *C. aaseri* SH14, was used for disruption of *CaAOX* genes [2]. This vector contains an autonomous replicating sequence and *NAT1* (NTC acetyl transferase) gene as a selection marker gene against NTC. The sgRNA sequences targeting for each *CaAOX* gene (*CaAOX2*: 5′-CTGCATTTCCGGCATTCCCA-3′, *CaAOX4*: 5′-GCACATGGTTCTAATGTTGC-3′, and *CaAOX5*: 5′-CCAACCCCAAGAAGCTACA-3′) were selected by online CRISPR gRNA design tool, ATUM (www.atum.bio/eCommerse/cas9/input). Target sequences with a low identities against *C. aaseri* genome database were selected. To construct double mutants by simultaneous disruption of *CaAOX* genes, sgRNA targeting sequences were selected from the conserved regions containing PAM sequence (NGG) (for *CaAOX2*/*CaAOX4*: 5′-CTCATATTGGAGCTACAAAA-3′, for *CaAOX2*/*CaAOX4*: 5′-GGTGCTAAAATGGGTAG AGA-3′)(Fig. S2). Each sgRNA was fused with translation elongation factor (*TEF1*) promoter, hammerhead ribozyme, and hepatitis delta virus ribozyme by polymerase chain reaction (PCR). The sgRNA expression cassettes were then cloned into the NotI-XbaI sites of pAN-Cas9 to make pAN-Cas9-gAOXn and confirmed by Sanger sequencing. All PCR amplifications were done using high-fidelity Pfu DNA polymerase. Primers used in this study were listed in Table S1.

### Transformation and Characterization of *C. aaseri* SH14 Transformants

Transformation of foreign genetic materials into *C. aaseri* SH14 was done using electroporation as described in previous study [2]. Competent cells were prepared in transformation buffer containing 5 mM lithium acetate, 0.5 M sorbitol, 10 mM Tris-HCl (pH 7.4), and 1 mM EDTA, and then 0.01 volume of freshly prepared 1 M dithiothreitol was added. After 1 h incubation at room temperature, cells were washed with 1 M sorbitol three times, and then resuspended in 0.5 ml of 1 M sorbitol for transformation. For each transformation, 0.5 μg of plasmid DNA was mixed with 100 μl of competent cells in a 2 mm electroporation cuvette before being incubated on ice for 10 min. Electroporation was performed using the Gene Pulser II (Bio-Rad, USA) at 2.25 kV, 50 μF, and 200 Ω. After electroporation, 1 ml of YPD containing 1 M sorbitol was added before incubation at 30°C overnight. All transformants were selected on YPD supplemented with 20 μg/ml NTC as selection media after a 3-day incubation at 30°C. Disruption of *AOX* genes was confirmed by colony PCR. Randomly selected colonies were suspended in lysis buffer (10 mM sodium phosphate, and 2 mg/ml lyticase) and incubated at 37°C for 30 min. Next, proteinase K solution (2 mg/ml) was added to the mixture, which was then incubated at 50°C for 10 min followed by inactivation of the proteinase K at 80°C for 10 min. Cell lysate (1 μl) was used as a template for PCR analysis. The mutations caused by CRISPR-Cas9 from each transformation were confirmed by direct sequencing of the PCR products by Bionics (Korea) without cloning into a plasmid vector.

### Expression and Characterization of AOX Genes in *C. aaseri* SH14

To use as an auxotrophic selection marker for the expression of *AOX* genes, *CaURA3* expression cassette containing promoter and terminator was amplified from *C. aaseri* SH14 genomic DNA by PCR, and cloned into the KpnI and XhoI site of pBluescript II vector, and the vector was named as p*CaURA3*. Open reading frame (ORF) of *CaAOX2*, *CaAOX4*, and *CaAOX5* gene were fused with 600 bp of *C. aaseri* glyceraldehyde 3-phosphate dehydrogenase promoter (GAPDHp), and 200 bp of GAPDH terminator (GAPDHt), and cloned into the XbaI, and XhoI site at pCaURA plasmid, respectively. Plasmids were linearized by XbaI and KpnI restriction enzymes to produce a fragment containing *CaURA3* and *AOX* expression cassette. *C. aaseri* SH14-245 (*ura3*, aox2, aox4, and aox5 mutant) strain was transformed with the linearized fragments. Integrations of *CaAOX* expression cassettes into the *C. aaseri* SH14 genome were confirmed by colony PCR using Gdh-F primer, and each AOXs-R internal primer, located at *CaAOX2*, *CaAOX4*, and *CaAOX5* ORF. To confirm the *CaAOX* gene expression, each cell was grown in 50 ml YPD broth for 48 h at 30°C. The cells were harvested by centrifugation at 10,000 ×*g* at 4°C for 30 min, and lysed using TRIzol^®^ reagent (Invitrogen) according to manufacturer’s instructions. Cell lysates were analyzed by SDS-PAGE using commercial NuPAGE Novex 4–12% Bis-Tris polyacrylamide gels (Invitrogen) and stained with Coomassie blue. Western blot analysis was performed using anti-His antibody (Sigma Chemicals Co.) after 1:1000 dilution. Protein samples were electrophoresed and then transferred to a nitrocellulose membrane using the iBot 2 Dry Blotting System (Thermo Fisher Scientific, USA) following the manufacturer’s instructions. The reacting antibodies were detected with anti-mouse immunoglobulins conjugated to alkaline phosphatase (Sigma Chemicals Co.) Acyl-CoA oxidase activity was determined by using the method describe by Wang *et al*. [22]. The tested acyl-CoA substrates were butyryl-CoA (C4:0-CoA), hexanoyl-CoA (C6:0-CoA), octanoyl-CoA (C8:0-CoA), decanoyl-CoA (C10:0-CoA), lauroyl-CoA (C12:0-CoA), myristoyl-CoA (C14:0-CoA), and palmitoyl-CoA (C16:0-CoA) (Sigma Chemicals Co.)

### Statistical Analyses

All data are presented as the mean value ± SD of three experiments. Statistical comparison of growth and ACOX activity were performed using Student’s *t*-test with a two-tailed distribution (Microsoft Excel), and compared to the appropriate control strain. Values were considered statistically significant at a *p*-value of < 0.05.

## Results and Discussions

### Construction of ACOX Isozyme Mutants

Three acyl-CoA oxidase genes found in *C. aaseri* SH14 genome were named as *CaAOX2*, *CaAOX4*, and *CaAOX5* based on the result of a homology search against the NCBI database using BLASTP program. The amino acid sequence of *CaAOX2* (55.17%) and *CaAOX4* (66.43%) showed the highest homology with ACOX2 and ACOX4 from *Candida tropicalis*. The amino acid sequence of *CaAOX5* showed the highest homology (57.37%) with ACOX5 from Candida albicans. Although we named the *CaAOX* genes according to homology analysis, identification of the functional differences among ACOXs using homology study is very difficult because there is no information on the residues that determine the substrate specificity, and the catalytic core regions are almost conserved (Fig. S1).

To identify the functional difference between the three *C. aaseri* SH14 ACOX isozymes, single, double, and triple mutant strains were constructed. Respective genes were inactivated by the CRISPR-Cas9 system that was developed for genome engineering of diploid yeast *C. aaseri* SH14. The Cas9 gene and sgRNA were expressed under the control of constitutive *TEF1* and *GAPDH* promoters, respectively, in an episomal vector, pANCas9sgRNA. The sgRNA target sequences for each isozyme were designed using the CRISPR gRNA design tool, ATUM (www.atum.bio/eCommerce/cas9/input). The highest scores from ATUM with fewer than 15 identities from BlastN analysis against *C. aaseri* genome database were selected as sgRNA target sequences. For single disruption, sgRNA specific to each isozyme was designed as sgAOX2, sgAOX4 and sgAOX5 (Fig. S2). The pANCas9 plasmids carrying different sgRNAs were transformed into the *C. aaseri* SH14 strain to create an indel mutation at the target sites. Mutations were confirmed by direct sequencing of the target locus amplified by PCR.

*CaAOX2*/*CaAOX4* and *CaAOX4*/*CaAOX5* double mutants (SH14-24 and SH14-45) were constructed at once by the transformation of pANCas9sgAOX24 and pANCas9sgAOX45 containing an sgRNA target sequence specific to *CaAOX2*/*CaAOX4* and *CaAOX4*/*CaAOX5*. *CaAOX2*/*CaAOX5* double mutant (SH14-25) was constructed by sequential disruption of *CaAOX2* and *CaAOX5* because there was no available sgRNA target sequence specific to *CaAOX2* and *CaAOX5* (Fig. S2). Mutation efficiencies in each case were higher than 40%. The triple mutant, SH14-245, was constructed in a previous study [2]. In this study, we created 6 mutants and their genotypes are listed in Table 1.

### Characterization of Mutant Strains

**Fig. 1:**
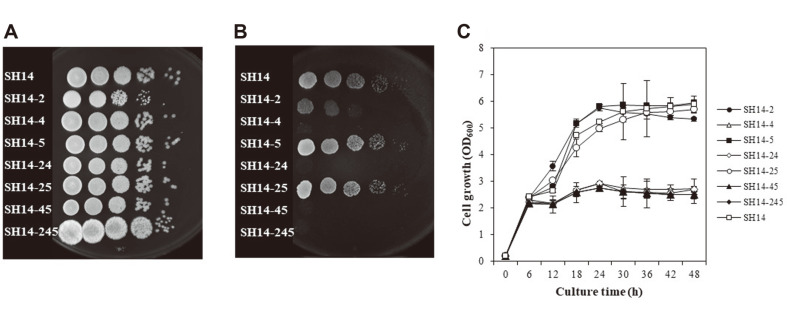
Growth of wild-type (SH14) and mutant strains on YPD and minimal medium supplemented with 1% oleic acid medium (SMO). Yeast strains grown in liquid YPD medium were collected, washed twice with sterile distilled water, and suspended to OD_600_ of 1.0. Serial dilution was prepared and 10 μl from each diluent was dotted onto YPD (**A**) and SMO (**B**) plates. The collected cells from overnight cultures were inoculated into SMO broth medium at an initial absorbance OD_600_ of 0.2 (**C**).

The involvement of acyl-CoA oxidase isozymes in peroxisomal β-oxidation was investigated by the evaluation of the growth of mutants on minimal media containing oleic acid as the sole carbon source. Serially diluted cells were dotted on YPD, and SMO plates (Fig. 1). There was no significant difference in growth between wild-type strain, SH14-2, SH14-5, and SH14-25 strain. Slightly lower growth of SH14-2 in SMO medium than wild-type strain was a result of a low number of dotted cells compared to other strains (Fig. 1A). On the other hand, none of the strains containing the *AOX4* mutation (SH14-4, SH14-24, SH14-45, and SH14-245) could grow on SMO medium (Fig. 1B). Moreover, similar results were observed in liquid culture (*p* < 0.05) (Fig. 1C).

To use long-chain fatty acid as the carbon source by β-oxidation, sequential reaction by the long chain-, medium chain-, and short chain-specific ACOX or an enzyme with broad substrate specificity is required. The *CaAOX2*, *CaAOX5* single mutants (SH14-2 and SH14-5), and double mutant (SH14-25) grew similarly to wild type in SMO medium due to the remaining *CaAOX4* isozyme (Fig. 1). On the other hand, despite *CaAOX2* and *CaAOX5* isozymes working normally, *CaAOX4* mutant (SH14-4) did not grow in SMO medium. These results imply that *CaAOX4* is an essential ACOX with broad substrate specificity for the utilization of long-chain fatty acids.

### Expression of ACOX Isozyme in *C. aaseri* SH14-245

**Fig. 2:**
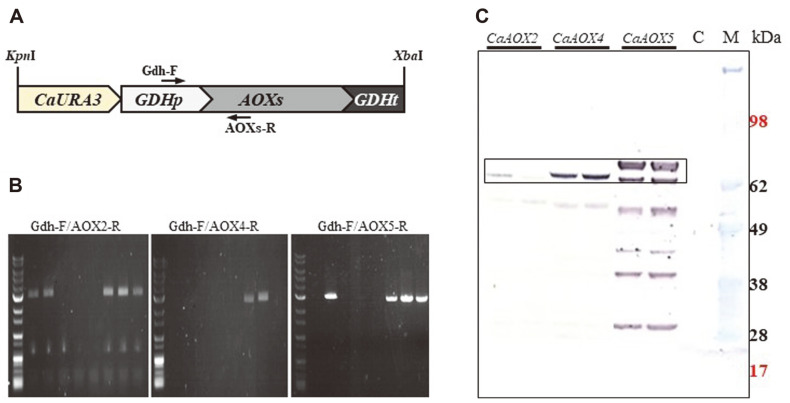
Expression and characterization of *CaAOX2*, *CaAOX4* and *CaAOX5* genes in the SH14-245 strain. Schematic structure of expression cassette (**A**), colony PCR of transformant (**B**) and western blot (**C**) of recombinant strains expressing *CaAOX2*, *CaAOX4*, and *CaAOX5* genes. Primers used in colony PCR were indicated by arrows. Intracellular fractions were analyzed using an anti-His antibody. C is the negative control (SH14-245). CaAox2p, CaAox4p CaAox5p bands are indicated by a box. M: protein size marker.

To study the substrate specificity of acyl-CoA oxidase isozymes, each isozyme was expressed in *C. aaseri* triple mutant (SH14-245). Since the transcription of *AOX* genes in yeast is repressed in glucose [23], for strong and stable expression of *AOX* genes, the ORFs of *CaAOX2*, *CaAOX4*, and *CaAOX5* were fused with constitutive *GAPDH* promoter and integrated into the genome of *C. aaseri* SH14-245 using *CaURA3* as selection marker. Transformants were verified by colony PCR and expression of ACOX was confirmed by western blot analysis of cell extract using an anti-His antibody. The expected sizes of *CaAOX2*, *CaAOX4*, and *CaAOX5* isozymes were 79.1 kDa, 79.6 kDa, and 80.2 kDa, respectively. In the western blot analysis, the expected size protein bands, with several smaller protein bands, were detected only in the transformants, indicating successful expression of the recombinant proteins (Fig. 2A). In addition, the smaller bands seem to be proteins degraded by endoprotease.

### Characterization of Recombinant ACOX Isozyme Produced in *C. aaseri* SH14

**Fig. 3:**
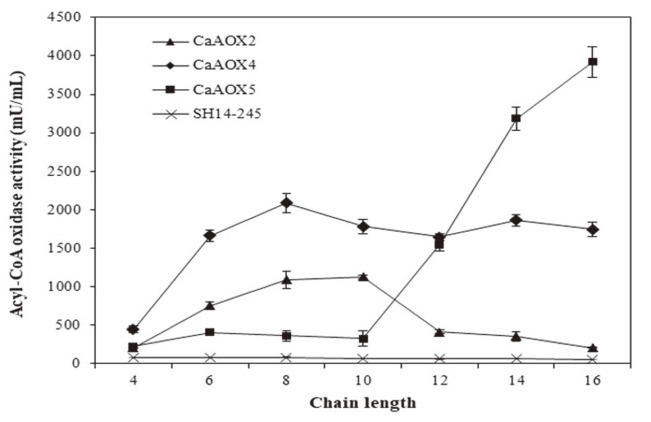
Characterization of substrate specificity of recombinant CaACOX. ACOX isozyme activity was measured independently using C6-C16 fatty acyl-CoA. Protein concentrations were standardized.

The activity of ACOX isozymes against fatty acids with chain lengths between 4 and 16 carbons was analyzed using the cell extract of transformants. As the SH14-245 strain cannot use long-chain fatty acid as a carbon source (Fig. 1), no ACOX activity was measured in the SH14-245 strain grown in a glucose medium. The absence of detectable ACOX activity in the SH14-245 strain means that there are no functional ACOX genes except *CaAOX2*, *CaAOX4*, and *CaAOX5*. *CaAOX5* isozyme showed substrate specificity toward long-chain fatty acid longer than C12. Meanwhile, *CaAOX2* isozyme preferred short- and medium-chain (C6-C12) fatty acids. *CaAOX4* isozyme showed broad–spectrum substrate specificity from C6-C16 (*p* < 0.05) (Fig. 3). Considering the result that *CaAOX4* mutant did not grow in SMO medium despite *CaAOX2* and *CaAOX5* isozyme working normally (Fig. 1), *CaAOX2* and *CaAOX5* isozymes were shown to be insufficient for complete β-oxidation of long-chain fatty acid, and *CaAOX4* isozyme was indispensable. Since the *CaAOX5* isozyme has substrate specificity toward long-chain fatty acid, the SH14-24 strain (*CaAOX2* and *CaAOX4* double mutant) could be used for bioconversion of long-chain fatty acid to medium-, and short-chain dicarboxylic acids such as sebacic acid (C10), suberic acid (C8), and adipic acid (C6) for polymer-based industries.

## Supplemental Materials

Supplementary data for this paper are available on-line only at http://jmb.or.kr.

## Figures and Tables

**Table 1:** *C. aaseri* SH14 AOX mutant strains constructed in this study.

Type	Strain	Genotype	Growth in SMO
Wild type	SH14	*ura3*	Normal
Single mutant	SH14-2	*ura3, aox2*	Normal
	SH14-4	*ura3, aox4*	None
	SH14-5	*ura3, aox5*	Normal
Double mutant	SH14-24	*ura3, aox2, aox4*	None
	SH14-45	*ura3, aox4, aox5*	None
	SH14-25	*ura3, aox2, aox5*	Normal
Triple mutant	SH14-245	*ura3, aox2, aox4, aox5*	None
