# Supplementary material for: Characterization of Acyl-CoA Oxidases from the Lipolytic Yeast Candida aaseri SH14
Source: J Microbiol Biotechnol. 2022 Jun 6;32(7):949–54. doi: 10.4014/jmb.2205.05029 (PMC9628930; doi:10.4014/jmb.2205.05029)
Supplement: Supplementary file 1 [file jmb-32-7-949-supple.pdf]

J. Microbiol. Biotechnol.
https://doi.org/10.4014/jmb.2205.05029

Supplementary Figure 1. Alignment of amino acid sequences of *CaAOX2, CaAOX4,* and *CaAOX5*.

```
                    *        20         *        40         *        60         *        80         *       100
AOX2 : -MFVTNKIDSSEAPRPSELISNERAAFPAPPRTKIHQFLEGSQKNSEIILKISSQLEKDPILQSSFGEYDMLTTQQREITAIRIDRLTNYREIESIDDFF :  99
AOX4 : MFNVSNSVDSLKGEDPKTLIATERAQQ-KWDPVIMNYFLEGSKETSEINKRLVQQMERDPIIRANPRFYDLSKPEQREITAIKIDRISQYLEQDSVEEYT :  99
AOX5 : -MNVNKVVSSSKPPTPRSYIQSERDAA-VFDPEIMNYFLEGSKERAEETKRMVQQMERDPILSARSNDYDMTKAESREATINKINRLSRYIEGETFDEFE :  98

                    *       120         *       140         *       160         *       180         *       200
AOX2 : TRINLITIYDPSIGIRISINLGLFINCIKGNGTASQVEYWCNRKEALILKQIYGCFAMTELGHGSNVSGCETTATFDQETDEFIINTPHIGATKWWIGGA : 199
AOX4 : RRSSLISVFDPQINVRIGVNLGLFIHCIRGNGTAEQLKFWSVTKEDKFLKGIYGCFAMTELAHGSNVAGLETTATFDKERDEFIINTPHIGATKWWIGGA : 199
AOX5 : RRMSLISVFDPQIHTRIGVHLGLFIGCIRGNGTFAQVNYWAIEKESAFIKNIYGCFAMTELAHGSNVAGLETTATFDEDNDQFIINTPHIGATKWWIGGA : 198

                    *       220         *       240         *       260         *       280         *       300
AOX2 : AHSATHSVVYARLLVHGKDYGVKTFIVPLRDSNHKLMPGVAIGDIGGKMGRSGVDNGWIQYSDVRIPRFFMLQKWCKVDRNGQVILPPLEQMSYISLLGG : 299
AOX4 : AHSATHTACYARLIVDGKDYGVKTFIVPLRDSNHKLMPGVTVGDIGAKMGRDGIDNGWIQFSSVRVPRFFMLQKFCKVSREGEVTLPPLEQLSYSALLGG : 299
AOX5 : AHSATHSTVYARLIVKGKDYGVKTFVVPLRDSNHDLMPGVVVGDIGAKMGRDGIDNGWIQFSSVRIPRFFMLQKYCKVSSDGEVTLPPNNQLSYGALLGG : 298

                    *       320         *       340         *       360         *       380         *       400
AOX2 : RVFMAVDSYRICARFITIATRYGVGRRQFKTDGDVES----QLLDYPLHQRRLMPFLALTYMMGVGTRRIMDSHKSIVELLDQAVAKNDKAGIITGLNDT : 395
AOX4 : RVTMVMDSYRISARFTTVALRYAIGRRQFKTDGPEET----QLLDYPLHQRRLLPYLALTYLISSGAYKIEATMDSVINTLDEAVVKNDMKTIMKSINDM : 395
AOX5 : RVVMVKDAFRWSSRAITIATRYGVGRKQFQAAKSKETDPENQIMNYPLHQRRLIPILALCYAFSNGANVIADYAKEANEILDSAVSNDDKAGIDKGIALI : 398

                    *       420         *       440         *       460         *       480         *       500
AOX2 : KSLFNDSGSLKATLTWFAADCIDQCRQACGGHGYSAYNGFGKSYDDWVVQCTWEGDNNVLSMSCGKTIINNMKLVLK-GKKITGTMDFLNSTK--IAEST : 492
AOX4 : KSLFIDSASLKSTCTWLAADCIDQCRQACGGHGYSAYNGFGKAFNDWVVQCTWEGDNNVIALSVGKPLIRSIDEIINKGKKASGSISFLNKVGDYLNENN : 495
AOX5 : KSLFIASGSLKSTCTWLTLNNIDQCRQACGGHGYSAYAGFGKAYNDFAVQCTWEGDNNILGMSVGKQLVKNIDNVLKGGEKPEGILAFLNDSKKYIDNKD : 498

                    *       520         *       540         *       560         *       580         *       600
AOX2 : AIIDSLDSLTAENVLILVQLLIIRVAIQTLEQMDNQKLKMEDVSYNRVVLSKLRCHHYLLQTFIQKLQQADS----EITDVISIVMRVYFLVYIIEDYSS : 588
AOX4 : VINSLDDVNNLEKNLLAIEVLIIRICKDSIDTIKESKGKYEVVSAAMVTISKLKAHHYLLTEFIGRLNSADG----SIKEYIELIAKLYTSSMIFDKFSG : 591
AOX5 : VVIKTEDISNSKKILEAIQVVLIRGSYCCLQTLKANGDDWDYIGADLVTLSKILAHFFLLSAFIDKVETMKSEEEKPLIPIIDKLAQLYSASIVIEQFSG : 598

                    *       620         *       640         *       660         *       680         *       700
AOX2 : LLIEHELITPSIKSKVSMKLVPQILQEMRQKSIVLTDSFLQPDNLIHSSIGKYNGDIYENYFNVVNQQNIA-FNTKAPYSEKFEAMLNRDSIEARERYEK : 687
AOX4 : DFLTYSVISPKLMGKINQVHIPKLCLEIRPLTVNYTDSFQQSDMLLNAPIGNYNGDIYENYFNVVKQENNP-FNTKAPYSTALEAMLNRSSLNDRERNEK : 690
AOX5 : FFLTYSVISADAMTTLTTGRIQDLCGQLRPHVIPLTDAFCLSDMLINSSLGGYDGNFYEEYFKIVKDNNPPPASYRASYTPAIMNMLHRPSYDDRTRQEF : 698

                    *
AOX2 : KE--ADV------- : 692
AOX4 : SEEVKSIISK---- : 700
AOX5 : SDEVADMIDPEAED : 712
```

Supplementary Figure 2. sgRNA target sequences for disruption of *CaAOX2, CaAOX4,* and *CaAOX5*.

PAM sites are indicated by red line

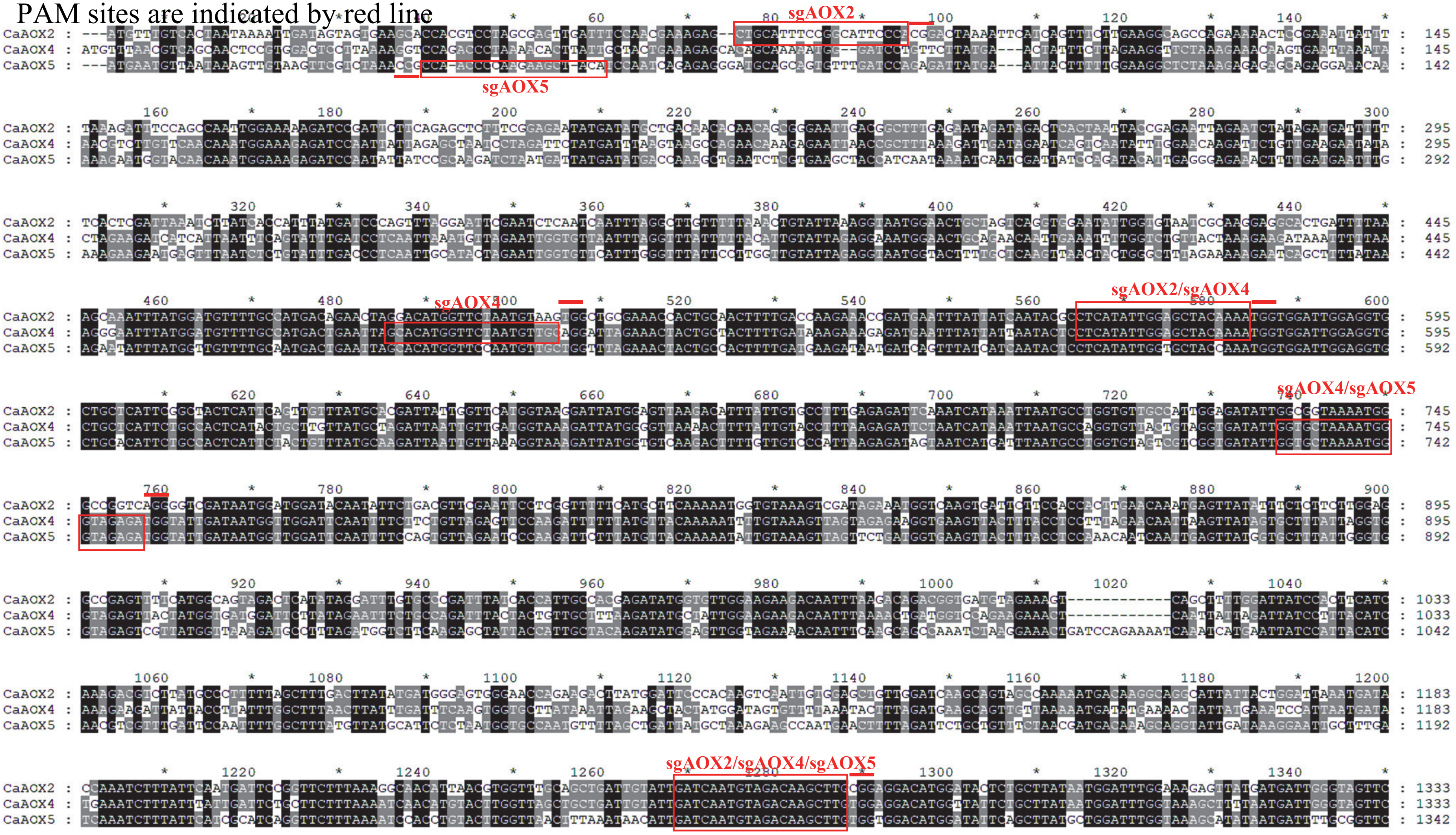

Supplementary Table S1. Primers used in this study

Primer	Sequence 5' – 3'	use
**GAPpF**	CCGCGGCCGCTCTAGAGTGTATAGTTGCTGG	Sense primer fot GAPDH promoter
**GAPtR**	ATCCTCGAGATATCGTAATATCGT	Antisense primer for GAPDH terminator
**URA3F**	TATCTCGAGGATATCCATTAATTC	Sense primer fot CaURA3 gene
**URA3R**	TTGGGTACCCCGTCATAAATTATC	Antisense primer for CaURA3 gene
**GAP-AOX2F**	TTTACAAGATTTAAAATGTTTGTCACTAAT	Sense primer fot CaAOX2 gene to fuse with GAPDH promoter
**AOX2-GAPpR**	ATTAGTGACAAACATTTTAAATCTTGTAAA	Antisense primer for GAPDH promoter to fuse with CaAOX2 gene
**AOX2HTF**	TGAAAAGAAAGAAGCTGATGTTCATCATCACCATCACCATTGAGTCGACCTAGCCGTGGAACAT	Sense primer fot GAPDH promoter to fuse with CaAOX2 gene
**AOX2HTR**	ATGTTCCACGGCTAGGTCGACTCAATGGTGATGGTGATGATGAACATCAGCTTCTTTCTTTTCA	Antisense primer for CaAOX2 gene to fuse with GAPDH promoter
**GAP-AOX4F**	TTTACAAGATTTAAAATGTTTAACGTCAGC	Sense primer fot CaAOX4 gene to fuse with GAPDH promoter
**AOX4-GAPR**	GCTGACGTTAAACATTTTAAATCTTGTAAA	Antisense primer for GAPDH promoter to fuse with CaAOX4 gene
**AOX4HTF**	AGTAAAGAGTATTTTAAGTAAACATCATCACCATCACCATTAGGTCGACCTAGCCGTGGAACAT	Sense primer fot GAPDH promoter to fuse with CaAOX4 gene
**AOX4HTR**	ATGTTCCACGGCTAGGTCGACCTAATGGTGATGGTGATGATGTTTACTTAAAATACTCTTTACT	Antisense primer for CaAOX4 gene to fuse with GAPDH promoter
**GAP-AOX5F**	TTTACAAGATTTAAAATGAATGTTAATAAA	Sense primer fot CaAOX5 gene to fuse with GAPDH promoter
**AOX5-GAPpR**	TTTATTAACATTCATTTTAAATCTTGTAAA	Antisense primer for GAPDH promoter to fuse with CaAOX5 gene
**AOX5HTF**	GATTGATCCTGAAGCAGAAGATCATCATCACCATCACCATTAAGTCGACCTAGCCGTGGAACAT	Sense primer fot GAPDH promoter to fuse with CaAOX5 gene
**AOX5HTR**	ATGTTCCACGGCTAGGTCGACTTAATGGTGATGGTGATGATGATCTTCTGCTTCAGGATCAATC	Antisense primer for CaAOX5 gene to fuse with GAPDH promoter
